# 

**Published:** 1894-04

**Authors:** 


### AMERICAN SURGICAL ASSOCIATION.

The American Surgical Association will meet in Washington, D. C., May 29th, 30th and 31st.

Special subjects for discussion:

1. "The Surgical Treatment of Empyema," by John Ashhurst, Jr., M. D. The discussion of the paper will be opened by Drs. N. P. Dandridge, C. B. Nancrede, T. F. Prewitt and DeF. Willard.

2. "Methods of Teaching Surgery," by J. S. Billings, M. D. The discussion of the paper will be opened by Drs. J. C. Warren, N. Senn, W. W. Keen, E. M. Moore, W. T. Briggs, and Hunter McGuire. It is desired that the discussion of this paper should be participated in by the Fellows generally.

3. "The Surgery of the Kidney," by L. M. Tiffany, M. D. The discussion of the paper will be opened by Drs. M. H. Richardson, H. H. Mudd, C. H. Mastin and Ford Thompson.

4. "Methods of Controlling Hemorrhage in Amputation at the Shoulder," as illustrated by three cases of amputation at the shoulder-joint and of the entire upper extremity, by W. W. Keen, M. D. The discussion of the paper will be opened by Drs. Roswell Park, C. B. Porter and J. William White.

5. Paper by Hunter McGuire, M. D.

6. Paper by Joseph Ransohoff, M. D.

Fellows who desire to present volunteer papers are requested to send the titles of the papers to the address of the Business Committee, 1429 Walnut Street, Philadelphia, not later than April 18, 1894.

The sessions of the Association will be held in the Lecture Room of the Medical Department of the Columbia College, 1325

H Street, N. W., Washington, D. C., on May 29th, 30th, 31st, and June 1st, from 10 A. M. to 1 P. M.

J. R. WEIST, M. D.,
Secretary.

---

ASSOCIATION OF GEORGIA CENTRAL RAILROAD SURGEONS.

The fourth annual meeting will be held at the Kimball House, Atlanta, Ga., April 17, 1894.

The following is the programme:

MORNING SESSION—10 A. M.

President's Address—Dr. A. C. North, Newnan, Ga.

Report of Committees.

Report of Treasurer.

New Business.

First Paper—"Anesthetics," Dr. W. B. Prather, Seale, Ala. Discussion.

Second Paper—"Medical Examination of Railway Employees." Dr. Ross P. Cox, Rome, Ga. Discussion.

Third Paper—"Treatment of Foreign Bodies in the Eye," Dr. A. W. Calhoun (Honorary member), Atlanta, Ga. Discussion.

Fourth Paper—"A Case of Tracheotomy," Dr. J. D. Dabney, Birmingham, Ala. Discussion.

Fifth Paper—"The Treatment of Wounds of the Scalp," Dr. J. I. Darby, Americus, Ga. Discussion.

Adjournment.

AFTERNOON SESSION—3 P. M.

Unfinished Business.

Informal Conference with Surgeons, Dr. W. H. Elliott, Chief Surgeon, Savannah, Ga.

Sixth Paper—"Dislocation of the Ankle Joint—a Case." Dr. H. A. Brown, Fort Valley, Ga. Discussion.

Seventh Paper—"Injuries of the Hand in Railroad Employees," Dr. R. H. Taylor, Griffin, Ga. Discussion.

Eighth Paper—"Comminuted Compound Fractures," Dr. Seth N. Jordan, Columbus, Ga. Discussion.

Ninth Paper—"The Surgery of the Spinal Cord, with Report of a Case," Dr. W. H. Hudson, LaFayette, Ala. Discussion.

Tenth Paper—"Four Years Work in Railway Surgery," Dr. Howard J. Williams, Macon, Ga. Discussion.

Election of officers.

Appointment of committees.

Adjournment.

### MEDICAL ASSOCIATION OF GEORGIA.

The forty-fifth annual session will be held in Atlanta, Ga., on April 18, 19 and 20, 1894.

### PROGRAM:

The President's Address, W. H. Elliott, M. D., Savannah.

Orator's Address, S. C. Benedict, M. D., Athens.

Trephining in Head Injuries with Paralysis of Opposite Arm, followed by Fungus Cerebri, R. M. Harbin, M. D., Calhoun.

The Treatment of Typhoid Fever, M. A. Clark, M. D., Barnesville.

Castration for Crime, J. G. Hopkins, M. D., Thomasville.

Drainage of the Peritoneal Cavity. The Use of the Syphon Pump, Wm. B. Gilmer, M. D., Macon.

Endometritis Cervical and Corporal Pathology, Symptomatology and Treatment, W. W. Stewart, M. D., Columbus.

Medical Legislation, A. C. Blain, M. D., Macon.

A Case of Sarcoma of Ileum following a Railway Injury, Howard J. Williams, M. D., Macon.

Teething, H. McHatton, M. D., Macon.

Treatment of Pneumonia, W. C. Humphries, M. D., Acworth.

Fluid Extract Jaborandi as an Abortive Treatment in Pneumonia, O. H. Buford, M. D., Cartersville.

Otorrhea, Dunbar Roy, M. D., Atlanta.

Phlegmasia Alba Dolens, Geo. H. Noble, M. D., Atlanta.

A Cataract, J. H. Shorter, M. D., Macon.

Cancer of the Uterus, the Remote Result of Operative Interference, W. A. Crow, M. D., Atlanta.

Treatment of Surgical Shock, W. F. Westmoreland, M. D., Atlanta.

The Time for Operation in Strangulated Hernia, W. P. Nicolson, M. D., Atlanta.

Operation for Fibro-Cystic-Sarcoma Involving the Right Inferior Maxillary Bone, J. McFadden Gaston, M. D., Atlanta.

"Diagnosis," H. Perdue, M. D., Barnesville.

Some Unusual Sequences of La Grippe, with Report of Cases, J. M. Head, M. D. Zebulon.

Treatment of Compound Fracture of Skull, with Report of Case Wm. S. Goldsmith, M. D., Altanta.

Treatment of Traumatic Epilepsy, with Report of Case, J. I Darby, M. D., Americus.

Pathology and Treatment of Pneumonia, J. C. Johnson, M. D., Atlanta.

Malignant Growths of the Skin; Diagnosis and Treatment, M. B. Hutchins, M. D., Atlanta.

Chronic Sore Legs and How to Cure Them, J. W. Hallum, M.D.

A Case of Gonorrhea in a Female, R. J. Nunn, M. D.

A Case of Extra-Uterine Pregnancy, John G. Earnest, M. D., Atlanta.

Suggestion and its Therapeutic Uses, Samuel C. Benedict, M. D., Athens.

An Operation for Hemorrhoids, R. H. Taylor, M. D.

Galvanism in Office Practice and in Gynecology, J. C. LeHardy, M. D.

Typhoid and Typho-Malarial Fevers, Their Differential Characteristic Features, A. A. Smith, M. D.

Foreign Bodies in Larynx, J. M. Hull, M. D.

Report of Case of Inflammation of External Auditory Canal Following Facial Erysipelas, P. R. Cortelyou, M. D., Marietta.

Primary and Diphtheritic Croup, the Operative and the Expectant or Medical Treatment, F. M. Ridley, M. D., LaGrange.

Sacrificial Surgery, Ovaries, Tubes and Uterus, Ross P. Cox, M.D.

Case Report, Extra-Uterine Superfœtation, R. R. Kime, M. D., Atlanta.

Some Remarks upon Appendicitis and Report of Cases, W. S. Elkin, M. D., Atlanta.

Rare Cases in Obstetrics, William F. Holt, M. D.

The Termination of Appendicitis when not Subjected to Operation, A. M. Cartledge, M. D.

The Tampon in Gynecology, James C. Avary, M. D., Atlanta.

The Recent Outbreak of Smallpox in Atlanta, Ga., James C. Avary, M. D., Atlanta.

Conjunctival Cysts in Each Eye, W. L. Bullard, M. D.

The Treatment of Corneal Ulcers with the Galvano-cautery, Arthur G. Hobbs, M. D., Atlanta.

Nasal Sprays Put to Use, George W. Brown, M. D., Atlanta.

Appendicitis, with Report of Cases, F. W. McRae, M.D., Atlanta.

Phlegmasia Alba Dolens, George H. Noble, M. D., Atlanta.

A Plea for the Closer Recognition of Dermatology, Bernard Wolff, M. D.

Suture of the Tendo Achillis, Report of a Case and Exhibition of Patient, L. B. Grandy, M. D., Atlanta.

Members of the profession cordially invited to attend.

W. H. ELLIOTT, M. D., President.

DAN. H. HOWELL, M. D., Secretary.

---

### AMERICAN MEDICAL ASSOCIATION.

*San Francisco Meeting, June* 5-8, 1894.

### SECTIONS ON SURGERY AND ANATOMY.

John B. Roberts, M. D., Philadelphia, Pa., Chairman; Floyd W. McRae, M. D., Atlanta, Ga., Secretary.

It is proposed to devote a portion of the time of this section to the systematic consideration of a few selected subjects, upon which papers, each not occupying more than ten minutes, will be read. It is hoped that speakers discussing these papers will confine their remarks to brief addresses of five minutes' length. The topics and papers to be presented are as follows:

I. *Malignant Growth.*—The Pathology of Malignant Growths: E. Laplace, Philadelphia, Pa. A Critique of the Sporozoan Theory of Malignant Neoplasms from a Micro-technical Standpoint: A. P. Ohlmacher, Chicago, Ills. Clinical Recognition of Malignancy in Tumors: C. A. Wheaton, St. Paul, Minn.; Henry W. Coe, Portland, Oregon. The Necessity of Early Surgical Interference in Malignant Tumors: R. A. McLean, San Francisco, Cal. The Value of Caustics in Malignant Growths: John Parmenter, Buffalo, New York. The Radical Cure of Malignant Tumors by Operation: J. H. Wythe, Oakland, Cal. The Value of Inoculation with Septic or Toxic Agents in the Treatment of Malignant Neoplasms: John A. Wyeth, New York, N. Y.

II. *Tubercular Disease of Joints.*—Early Symptoms and Diagnosis of Tubercular Joint Disease: Emmet Rixford, San Francisco, Cal.; A. B. Judson, New York, N. Y. Conservative Treatment of Tubercular Joints: R. H. Sayre, New York, N. Y.; Harry M. Sherman, San Francisco, Cal.; James E. Thompson, Galveston, Texas. Operative Treatment of Tubercular Joints: Robert W. Lovett, Boston, Mass. Treatment of Tubercular Joints by Injections of Iodoform: N. Senn, Chicago. Treatment of Tubercular Joints by Injection, of Corrosive Sublimate: R. H. Plummer, San Francisco, Cal.

III. *Hernia.*—The Causation and Prevention of Hernia: James

T. Jelks, Hot Springs, Arkansas; C. M. Richter, San Francisco, Cal. The Management of Reducible Hernia: Emory Lanphear, Kansas City, Mo.; C. M. Fenn, San Diego, Cal. The Treatment of Irreducible Hernia: James B. Eagleson, Seattle, Washington. The Treatment of Strangulated Hernia: Joseph Ransohoff, Cincinnati, Ohio. The Radical Cure of Hernia: W. E. S. Davis, Birmingham, Ala.; H. O. Marcy, Boston, Mass.

IV. *Hemorrhoids, Fistule and Fissure.*—The Pathology and Symptomatology of Hemorrhoids, Anal Fistule and Anal Fissure: J. M. Matthews, Louisville, Ky.; David Powell, Maysville, Cal. Treatment of Hemorrhoids: H. M. Bishop, Los Angeles, Cal.; Charles B. Kelsey, New York, N. Y. Treatment of Anal Fistule: J. McF. Gaston, Atlanta, Ga.; G. B. Somers, San Francisco, Cal. Treatment of Anal Fissure: Thomas W. Huntington, Sacramento, Cal.; Lewis H. Adler, Jr., Philadelphia, Pa.

V. *Fractures.*—Treatment of Fractures of the Lower End of the Humerus: Oscar H. Allis, Philadelphia, Pa. Treatment of Fractures of the Lower End of the Radius: P. T. Connor, Cincinnati, Ohio; C. L. Bower, Philadelphia, Pa. Treatment of Fractures of the Neck of the Femur: Bedford Brown, Alexandria, Virginia. Treatment of Gunshot Fractures: George A. Goodfellow, Tucson, Arizona. Treatment of Fractures of the Shaft of the Femur: Llewellyn Eliot, Washington, D. C. Treatment of Open or Compound Fractures: H. H. Mudd, St. Louis, Mo.; John B. Hamilton, Chicago, Ills.

VI. *Obstruction to Urination in the Male.*—Effects of Obstruction in Urination upon the Bladder and Kidneys: J. William White, Philadelphia, Pa. Diagnosis and Treatment of Enlargement of the Prostate Gland: Hunter McGuire, Richmond, Va.; Wm. T. Belfield, Chicago, Ills. Symptoms and Treatment of Stone in the Bladder, William T. Briggs, Nashville, Tenn. Symptoms and Treatment of Tumors of the Bladder: John B. Deaver, Philadelphia, Pa.; C. F. Buckley, San Francisco, Cal. Treatment of Stricture of the Urethra: J. Rosenstirn, San Francisco, Cal.

Members who have specimens or patients to exhibit bearing on these topics, or who wish to make remarks in the discussion of them, are cordially invited to be present during the meetings of the Section. The titles of other papers to be presented to the Section will be published when the programme of the meeting of the Association is issued by the Committee of Arrangements.

JOHN B. ROBERTS,
Chairman Section on Surgery and Anatomy.
1627 *Walnut Street, Philadelphia, Pa.*

